# Pathogenesis of adherent-invasive *Escherichia coli* LF82 in human colonic epithelium is characterized by adhesive biofilms, mucus penetration, and contact-dependent cytotoxicity

**DOI:** 10.1080/19490976.2025.2573046

**Published:** 2025-10-29

**Authors:** Bethan Fay Evans, Tshering Dorji, Damira Bigaliyeva, Simon Chan, Stephanie Schüller

**Affiliations:** a Metabolic Health Research Centre, Norwich Medical School, University of East Anglia, UK; b Centre for Microbial Interactions, Norwich Research Park, Norwich, UK

**Keywords:** Crohn's disease, adherent-invasive *E. coli*, pathogenesis, colonic epithelium, colonoids, biofilm, mucus, cytotoxicity

## Abstract

Adherent-invasive *Escherichia coli* (AIEC) associated with Crohn's disease (CD) are traditionally defined by the adherence and invasion of epithelial cells and survival in macrophages. However, their interactions with differentiated intestinal epithelia remain largely unexplored. Here, we investigated the pathogenesis of AIEC prototype strain LF82 in polarized human colon carcinoma cells and colonic organoids. While LF82 infection of Caco-2 and T84 cells was characterized by CEACAM6-independent adherence, biofilm formation, inflammation, and contact-mediated cytotoxicity, invasion was comparably low to that of noninvasive *E. coli* MG1655. An investigation of additional AIEC isolates revealed that biofilm production and cell damage were specific for strain LF82. Infection of human colonoids confirmed biofilm formation, negligible invasion, and cytotoxicity of AIEC LF82. However, bacteria adhered preferentially to the mucus layer and penetrated to the epithelial surface. Our results suggest that LF82 pathogenesis in the human colon is characterized by the formation of adherent biofilms, mucus penetration, and contact-dependent cytotoxicity, which likely contributes to epithelial leakage and inflammation in CD.

## Introduction

Inflammatory bowel disease (IBD) is a chronic intestinal disorder affecting more than 3 million people in the USA and Europe. It includes two major forms, Crohn's disease (CD) and ulcerative colitis, and its incidence is increasing, particularly in newly industrialized countries.^1^ Research suggests that IBD is caused by a combination of environmental and genetic factors that result in microbial dysbiosis, a leaky gut epithelium and an overactive inflammatory immune response.^2^ Notably, a particular type of *Escherichia coli*, named adherent-invasive *E. coli* (AIEC), is highly prevalent in CD tissue, but it remains unclear whether AIEC is a cause or consequence of the disease.^3^ Therefore, it is important to understand the interactions between AIEC and the human intestinal epithelium.

The definition of AIEC pathogenesis is complicated by its genomic heterogeneity and lack of specific virulence markers.^4^ However, there is a common phenotype which is characterized by adhesion and invasion of epithelial cells and intracellular survival in macrophages.^5^^,^^6^ Adherence has been shown to be mediated by binding of the fimbrial adhesin FimH to the mannosylated host cell receptor CEACAM6, which is highly expressed in inflamed ileal tissue.^7^ In addition, the AIEC outer membrane proteins ChiA and OmpA facilitate intestinal epithelial binding by interacting with CHI3L1 and Gp96 glycoproteins, respectively.^8^^,^^9^ Notably, biofilm formation has been demonstrated for several AIEC strains and is considered a key virulence feature.^10^ In contrast to adherence, epithelial AIEC invasion is less well characterized, and findings rely predominantly on studies in nonpolarized cells.^5^^,^^11^ Notably, AIEC translocation across M cells has been demonstrated in ileal tissues.^12^^,^^13^ Furthermore, AIEC infection causes loss of intestinal epithelial barrier function by disrupting tight junction complexes^14–16^ and enhances the secretion of proinflammatory cytokines^17^^,^^18^ which are also prominent features in CD.^1^

Importantly, most of our current understanding of AIEC pathogenesis has been gained from studies in undifferentiated epithelial cell lines or susceptible mouse models, including transgenic mice expressing CEACAM6.^7^^,^^18^ To determine the relevance of these findings in conditions more relevant to the human gut, we have investigated the interactions of AIEC with differentiated human colonic cell lines and adult stem cell-derived organoids.

## Materials & methods

### Bacterial strains and culture conditions

The *E. coli* isolates used in this study are listed in Table 1. For infections, strains were grown standing in LB Lennox broth overnight at 37 °C. Growth curves were generated by diluting overnight cultures to OD_600_ 0.1 in Dulbecco's modified Eagle's medium/nutrient F-12 Ham 1:1 (DMEM/F12) medium (Gibco) in 96-well plates and monitoring OD_600_ in a FLUOstar Optima microplate reader (BMG) at 37 °C.

**Table 1.** *E. coli* strains used in this study.

*E. coli* strain	Pathotype	Description	Source/reference
LF82	AIEC	Ileal CD isolate	[5]
LF82 Δ*tssE1*	AIEC	LF82 with 341 bp deletion in *tssE1*	[19]
LF82 Δ*tssE3*	AIEC	LF82 with 396 bp deletion in *tssE3*	[19]
LF82 Δ*tssE1* Δ*tssE3*	AIEC	LF82 with deletions in *tssE1* and *tssE3*	[19]
NRG857c	AIEC	Ileal CD isolate	[20]
HM615	AIEC	Colonic CD isolate	[21]
HM605	AIEC	Colonic CD isolate	[21]
HM580	AIEC	Colonic CD isolate	[21]
H10407	ETEC	Human diarrheal isolate	[22]
MG1655	Lab strain	*E. coli* K-12 derivative	[23]

### Cell culture

T84 human colon carcinoma cells (ECACC 88021101) were cultured in DMEM/F12 medium (Gibco) supplemented with 10% fetal bovine serum and 2.5 mM L-glutamine (Merck). Caco-2 (ECACC 86010202) and LS174T human colon carcinoma cells (ECACC 87060401) were grown in DMEM medium (high glucose, Merck) supplemented with 10% fetal bovine serum, 4 mM L-glutamine, and nonessential amino acids (1×). To establish confluent monolayers, cells were seeded at a density of 1.2 × 10^5^ cells/well (T84) and 1 × 10^5^ cells/well (Caco-2, LS174T) in 24-well plates, and grown until fully confluent (~7 d). For polarisation, 5 × 10^5^ T84 cells were seeded on polyester Transwell filter inserts (12 mm diameter, 0.4 µm pore; Corning Costar) coated with type I rat tail collagen. To determine bacterial invasion from the apical or basal cell surface, 1.7 × 10^5^ T84 or 6.7 × 10^4^ Caco-2 cells were seeded on 6.5 mm diameter Transwell inserts with 3 μm pores. Transepithelial electrical resistance (TEER) was monitored using an STX electrode and an EVOM2 resistance meter (WPI), and values of >1500 Ω cm^2^ after 7–10 d of culture indicated the establishment of epithelial barrier function. The cells were grown at 37 °C in a 5% CO_2_ atmosphere.

### Human colonoid culture

This study was performed with approval from the University of East Anglia Faculty of Medicine and Health Sciences Research Ethics Subcommittee (Application ETH2122-1185). The samples were collected by the Norwich Research Park Biorepository (REC reference 19/EE/0089). Tissue samples from the transverse colon were obtained with informed consent from hemicolectomy resections or endoscopic biopsy specimens (Table 2). Colonoids were established as described previously^24^ with the following modifications. Colonic crypts were dissociated by incubation of tissue fragments in 2.5 mM EDTA (Fisher Scientific) in cold chelating solution for 30 min at 4 °C. Washed crypt pellets were seeded in Cultrex Basement Membrane Extract (Bio-Techne) and grown in expansion medium composed of Advanced DMEM/F-12 (Life Technologies) supplemented with 10 mM HEPES (Life Technologies), 2 mM GlutaMAX (Life Technologies), 50% (v/v) Wnt3a-conditioned medium, 20% (v/v) R-spondin-1-conditioned medium, 10% (v/v) Noggin-conditioned medium,^25^ 1× B27 supplement (Life Technologies), 1 mM N-acetylcysteine (Sigma-Aldrich), 50 ng/ml human epidermal growth factor (Life Technologies), 10 nM [Leu-15] gastrin (AnaSpec), 500 nM A83-01 (Tocris), 10 μM SB202190 (Stemcell Technologies), 1 μM prostaglandin E2 (Stemcell Technologies), and 10 μM Y-27632 (Tocris). For incubation with bacteria, fragmented colonoids were seeded on Transwell inserts (6.5 mm diameter, 0.4 µm pores, Corning Costar) coated with human type IV collagen (Sigma-Aldrich) (10 μg/cm^2^) and grown in expansion medium until confluent (5–7 d). Colonoid monolayers were subsequently differentiated by withdrawal of SB202190, Wnt3a, and R-spondin-1 for 4 d. Differentiation was confirmed by an increase in TEER.

**Table 2.** Patient characteristics of transverse colonoids established in this study.

Designation	Age	Sex	Diagnosis
TCC-2	53	Male	CD in descending colon
TCC-6	71	Male	IIeal CD, previous right hemicolectomy
TCC-7	20	Female	Colonic CD
TCN-1	60	Male	No pathology
TCN-2	74	Male	Adenocarcinoma in ascending colon
TCN-4	53	Female	Cecal cancer
TCN-5	66	Male	Cecal cancer

### Quantification of bacterial adherence, invasion, and intracellular replication

Cells were inoculated with 10 µl of bacterial overnight cultures (reflecting an approximate multiplicity of infection of 10), and gentamicin protection assays were performed as described previously.^5^ Briefly, the cells were incubated for 3 h at 37 °C in a 5% CO_2_ atmosphere. After the removal of nonadherent bacteria by three washes with PBS, which also detached the outer but not inner mucus layer of the colonoids, the cells were lysed with 1% (v/v) Triton X-100. Dilutions of the cell lysates were plated on LB agar, and colony-forming units (CFUs) were counted to quantify the number of adherent bacteria. To evaluate invasion and intracellular replication, cell culture medium containing 50 μg/ml gentamicin was added to kill the extracellular bacteria, and the plates were incubated for a further 1 h or 21 h, respectively. Bacterial adhesion, invasion, and replication counts were standardized to a bacterial inoculum of 10^7^ to account for differences in concentrations of overnight cultures between AIEC strains, which ranged from 0.7 to 2.0 × 10^9^ CFU/ml. To block type I fimbrial binding, 0.5% (w/v) D-mannose (Merck) was added to the initial inoculum. To inhibit CEACAM binding, the cells were incubated with anti-CEACAM1 (sc-166453, Santa Cruz), anti-CEACAM5 (sc-23928, Santa Cruz), anti-CEACAM6 (sc-59899, Santa Cruz; 17169525, Invitrogen), and anti-CEACAM7 (sc-59946, Santa Cruz) at a dilution of 1:100 for 1 h before bacterial inoculation. To block bacterial invasion, the cells were preincubated for 30 min with 1 μg/ml cytochalasin D (Enzo) or 0.5 μg/ml colchicine (Acros Organics).

### Immunofluorescence staining and microscopy

The cells were fixed in 3.7% formaldehyde (v/v) for 10 min at room temperature or in methanol/acetone (1:1) for 4 min on ice for mucin staining. The samples were blocked and permeabilized in 0.5% bovine serum albumin (w/v) and 0.1% Triton X-100 (v/v) in PBS for 20 min. The specimens were incubated in primary antibodies for 60 min at room temperature. The following antibodies were used in this study: goat anti-*E. coli* (1:400, ab13627, Abcam), rabbit anti-*E. coli* O, K antigens (1:400, E3500-06C, USBiologicals), rabbit anti-MUC2 (1:250, sc-15334, Santa Cruz), or mouse anti-CEACAM6 (1:200, sc-59899, Santa Cruz). For detection, the samples were incubated in donkey anti-mouse, rabbit or goat IgG conjugated with Alexa Fluor 488, 568 or 647 (1:400, Invitrogen) for 30 min. Cellulose, filamentous actin, and DNA were stained with calcofluor white (0.002% w/v, Merck), fluorescein isothiocyanate- or Alexa Fluor 647-conjugated phalloidin and 4ʹ,6-diamidino-2-phenylindole (DAPI, Merck), respectively. The samples were mounted in Vectashield (Vector laboratories) and analyzed using a Leica fluorescent light or LSM800 confocal laser-scanning microscope (Zeiss). MUC2 staining on colonoids was quantified with ImageJ.

### Biofilm formation

Biofilm formation was assessed by crystal violet staining.^26^ Overnight bacterial cultures were diluted to OD_600_ 0.01 in cell culture medium in 96-well plates and incubated at 37 °C for 24 h. The biofilms were stained with 0.1% (w/v) crystal violet in ethanol for 10 min. After drying, the stain was solubilized in 30% (v/v) acetic acid, and the absorbance was measured at OD_595_.

### Yeast agglutination

FimH expression of AIEC isolates was evaluated by yeast agglutination. Bacteria incubated in cell culture medium for 3 h were suspended in PBS at OD_600_ 0.5 and serially diluted in 96-well plates. Baker's yeast (20 mg/ml) was added at a ratio of 1:1, and agglutination was assessed after 20 min.

### Quantification of cytotoxicity

Cytotoxicity was assessed by staining with Trypan blue, which is only taken up by dead cells. After removal of the medium, the cells subjected to the respective treatments were incubated with 0.05% (w/v) Trypan blue for 15 min at 37 °C. Unbound dye was removed by washing with PBS, and monolayer integrity was confirmed by microscopy. The internalized dye was subsequently released by cell lysis in 1% (w/v) SDS, and the absorbance was determined at OD_590_.

### Cytokine secretion

Cytokines in cell supernatants were quantified using human IL-6, IL-1β, and TNF-α uncoated ELISA kits (Invitrogen) and a human IL-8 ELISA kit (PeproTech) according to the manufacturers' instructions.

### Statistical analysis

The data were analyzed with GraphPad prism version 10.4.2. The statistical tests applied are specified in the figure legends, and a *p-*value of <0.05 was considered statistically significant. The data are shown as individual data points with means and standard deviations.

## Results

### AIEC LF82 adherence but not invasion in colon carcinoma cells is higher compared with noninvasive *E. coli*

**Figure 1.**
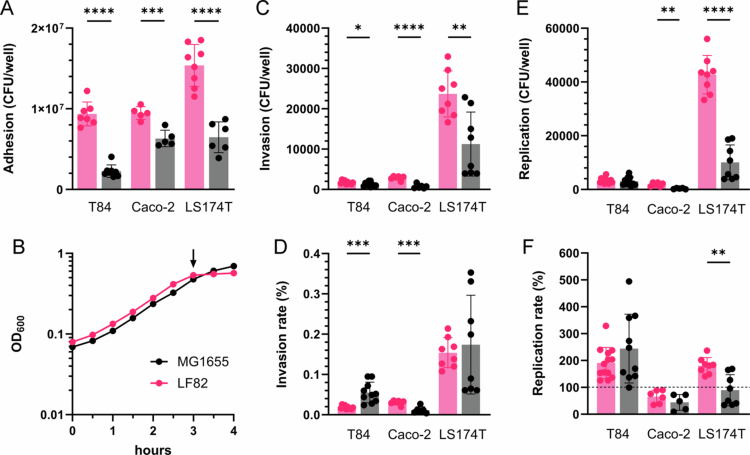
Adherence, invasion, and intracellular survival of AIEC LF82 in Caco-2, T84, and LS174T cells. Confluent cell monolayers were incubated with AIEC LF82 or noninvasive *E. coli* MG1655 for 3 h, and the number of adherent bacteria was determined by CFUs (A). In addition, bacterial growth in media was quantified by the OD_600._ The 3 h timepoint is labeled with an arrow (B). Invasion (C) and intracellular replication (E) were evaluated by killing extracellular bacteria with gentamicin and plating out cell lysates after 1 and 21 h of incubation, respectively. The results were normalized to an inoculum of 10^7^ bacteria. Invasion (D) and replication rates (F) were calculated relative to the total number of adherent and invaded bacteria, respectively. Significance was calculated using student's unpaired *t*-test (*p < 0.05, **p < 0.01, ***p < 0.001, and ****p < 0.0001).

Initial studies were performed using AIEC prototype strain LF82 isolated from the ileal mucosa of a CD patient^27^ and noninvasive *E. coli* K-12 derivative MG1655 as a control. Using a standard gentamicin protection assay^5^, adhesion, invasion, and intracellular survival were determined in the enterocyte-derived colon carcinoma cell lines Caco-2 and T84 and mucus-producing goblet cell-like LS174T adenocarcinoma cells^28^. While T84 cells form highly polarized columnar epithelia with structural similarity to colonic crypt cells^29^, Caco-2 cells more resemble enterocytes from the small intestine with respect to morphology and function^30^. As shown in Figure 1A, the adherence of AIEC LF82 to all the cell lines was significantly higher compared to MG1655. This was unrelated to differences in bacterial growth, as both strains showed similar kinetics over the 3 h infection period (Figure 1B). Similar to adhesion, a higher number of invasive bacteria was detected for LF82 versus MG1655 after 1 h of gentamicin treatment (Figure 1C). However, when this was normalized to the number of adherent bacteria, LF82 demonstrated a higher invasion rate in Caco-2 cells, whereas MG1655 was more invasive in T84 cells (Figure 1D). Notably, invasion rates were very low (≤0.05%). In LS174T cells, invasion rates were higher (~0.17%) and similar between both strains. After 24 h of incubation, LF82 showed a higher number of intracellular bacteria in Caco-2 and LS174T cells, while there was no significant difference between strains in T84 cells (Figure 1E). After normalization to the number of invasive bacteria, the replication rate of LF82 was higher in LS174T cells only, whereas no difference between strains was evident in T84 and Caco-2 cells (Figure 1F). Interestingly, both *E. coli* strains multiplied in T84 cells, while bacterial clearance was observed in Caco-2 cells.

**Figure 2.**
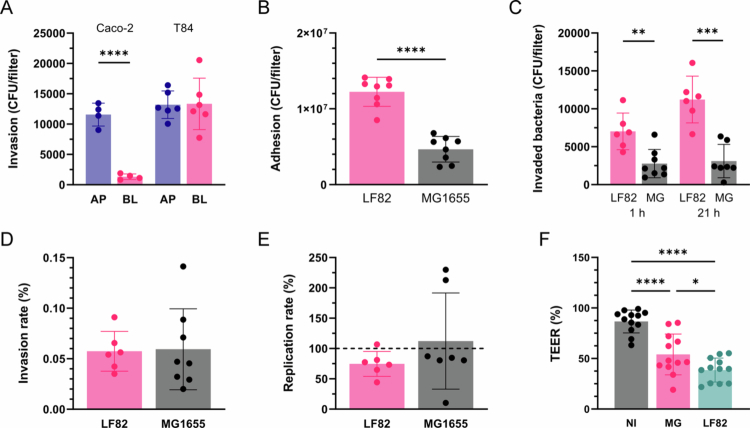
Interaction of LF82 with cells polarized on Transwell filters. (A) Polarized Caco-2 and T84 cells were infected with LF82 on the apical (AP) or basolateral (BL) side for 3 h, followed by 1 h of gentamicin treatment. The number of intracellular bacteria was determined by CFUs. (B–F) Polarized T84 cells were incubated with LF82, MG1655 on the apical side or left noninfected (NI) for 3 h followed by 1 or 21 h of gentamicin treatment. The number of adherent (B), intracellular bacteria (C), and the invasion and replication rates (D, E) were determined as described in Figure 1. In addition, TEER was measured after 21 h of gentamicin treatment and expressed as percentage relative to barrier function before incubation (F). Significance was calculated using student's unpaired t test (A‒E) or one-way ANOVA with Tukey's post hoc test comparison (F) (*p < 0.05, **p < 0.01, ***p < 0.001, and ****p < 0.0001).

As invasion rates of LF82 were negligible in Caco-2 and T84 cells, we determined whether cell entry was facilitated by bacterial exposure to the basolateral side of the epithelium. To this aim, Caco-2 and T84 cells were grown and polarized on Transwell inserts. As shown in Figure 2A, invasion of LF82 into Caco-2 cells occurred predominantly via the apical surface, whereas the number of intracellular bacteria was similar in apically and basolaterally infected T84 cells. Total apical adhesion, invasion, and intracellular replication of LF82 in polarized T84 cells were significantly higher compared with control strain MG1655 (Figure 2B and C). However, the invasion and replication rates of both strains after normalization were comparable (Figure 2D and E). In addition, incubation with both strains resulted in a significant reduction of epithelial barrier function, which was more pronounced during LF82 infection (Figure 2F).

### AIEC LF82 binding to colonic cell lines is characterized by biofilm formation and independent of CEACAM6

**Figure 3.**
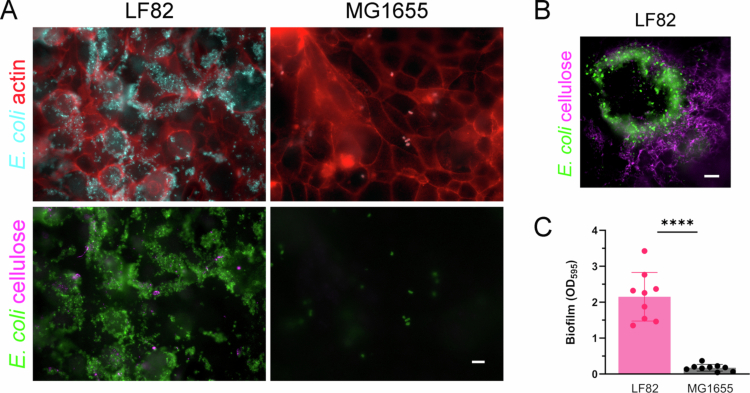
AIEC LF82 forms biofilms on human colonic cells. (A) Confluent Caco-2 cells were incubated with LF82, MG1655, or left noninfected (NI) for 3 h and stained for *E. coli* and actin (upper panels) or cellulose (lower panels). (B) LF82 biofilm formation on T84 cells shown at higher magnification. Representative images of *n* = 3 are shown. Bar = 5 µm. (C) Biofilm formation on 96-well plates was quantified after 24 h by crystal violet staining. Significance was calculated using student's unpaired *t*-test (****p < 0.0001).

**Figure 4.**
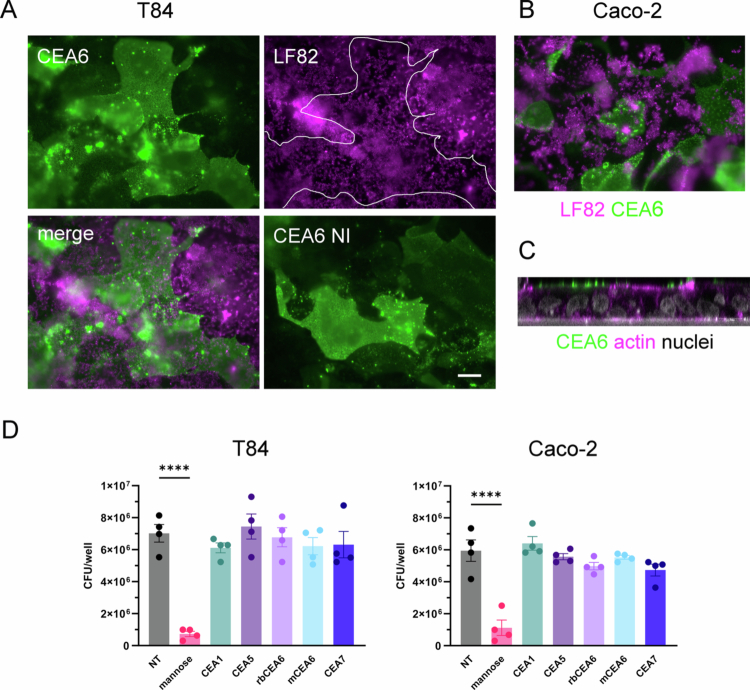
The binding of AIEC LF82 to colon carcinoma cells is mannose-dependent but not mediated by CEACAM6. Confluent T84 or Caco-2 cells were incubated with LF82 for 3 h and stained for *E. coli* (magenta) and CEACAM6 (green). (A) Staining of T84 cells is displayed as separate channels (CEA6, LF82) and a merged image. Cells with high CEACAM6 expression are outlined in white. In addition, CEACAM6 staining of noninfected cells is shown (CEA6 NI). (B) Merged channel image of Caco-2 cells. (C) XZ scan of T84 cells stained for CEACAM6 (green), actin (magenta), and cell nuclei (white). Scale bar = 10 µm. (D) Cells were preincubated with antibodies against CEACAM1, CEACAM5, CEACAM6, and CEACAM7 or left untreated (NT). For CEACAM6, a mouse (m) or rabbit (rb) antibody was used. The cells were infected with LF82 for 3 h, and adhesion was quantified by CFUs. Mannose was included to block fimbrial binding. Significance was calculated using one-way ANOVA with Dunnett's post hoc test for comparison to NT (****p < 0.0001).

We next visualized the adherence of *E. coli* strains LF82 and MG1655 to confluent Caco-2 and T84 cells by immunofluorescence staining. While few adherent bacteria were observed for MG1655, LF82 formed extensive biofilm-like structures on Caco-2 (Figure 3A) and T84 cells (data not shown). Biofilm formation was confirmed by the staining of cellulose fibers between adherent bacteria (Figure 3A and B). Epithelial LF82 biofilm formation was also reproduced on plastic surfaces (Figure 3C). To determine whether LF82 binding to Caco-2 and T84 cells was dependent on CEACAM6, which has been identified as a receptor for LF82 on ileal enterocytes,^7^ immunostaining and antibody blocking experiments were performed. As shown in Figure 4A and B, CEACAM6 was heterogeneously expressed on confluent T84 and Caco-2 cells. Confocal XZ scanning demonstrated that CEACAM6 was localized at the apical cell membrane (Figure 4C). Notably, some cells showed strong CEACAM6 surface staining, while others were devoid of receptor expression (Figure 4A and B). However, LF82 binding was not correlated with CEACAM6 expression patterns, and bacteria did not bind preferentially to cells with high CEACAM abundance (Figure 4A and B). While the addition of mannose significantly blocked AIEC LF82 binding, antibodies against CEACAM6 and additional colonic CEACAMs (CEACAM1, 5, and 7) did not inhibit bacterial adhesion (Figure 4D). In contrast, the adhesion of CEACAM6-binding enterotoxigenic *E. coli* H10407^31^ to T84 cells was significantly reduced in the presence of CEACAM6-specific antibodies (Figure S1).

### Infection with LF82 causes contact-dependent cell death and secretion of proinflammatory cytokines

**Figure 5.**
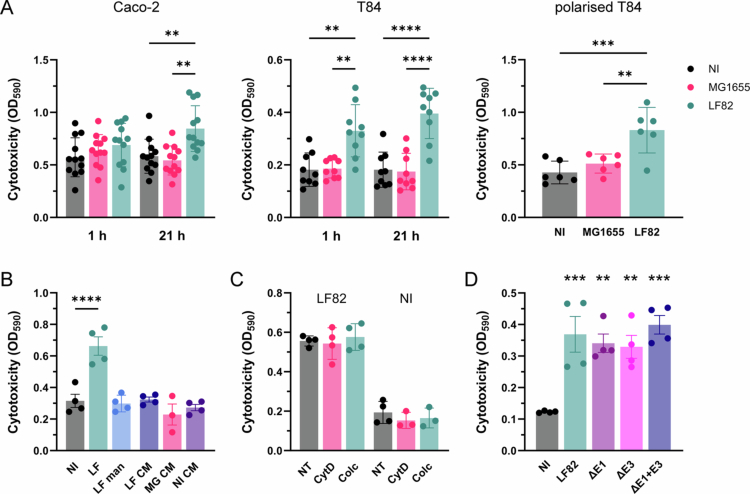
AIEC LF82 exhibits cytotoxicity to colon carcinoma cells which is contact-dependent but is not mediated by invasion or type VI secretion. (A) Confluent Caco-2 and T84 cells and polarized T84 cells grown on Transwell filters were incubated with AIEC LF82, *E. coli* MG1655 or left noninfected (NI) for 3 h followed by 1 h (confluent Caco-2 and T84 cells) and 21 h of gentamicin treatment. (B) T84 cells were incubated with AIEC LF82 in the presence (LF man) or absence of mannose (LF) or left uninfected for 3 h plus 21 h gentamicin treatment. In addition, conditioned media (CM) from cells incubated with strains LF82 (LF), MG1655 (MG) or left noninfected for 3 h were collected and added to fresh T84 cells for 21 h. (C) T84 cells were incubated with AIEC LF82 for 3 h plus 21 h gentamicin treatment in the presence or absence (NT) of cytochalasin D (CytD) or colchicine (Colc). Noninfected controls NI containing both drugs were included. (D) T84 cells were incubated with wild-type AIEC LF82 or type VI secretion deletion mutants in *tssE1* (ΔE1), *tssE3* (ΔE3) or both (ΔE1 + E3) or left noninfected (NI) for 3 h followed by 21 h of gentamicin treatment. Dead cells were subsequently stained with Trypan blue, and dye uptake was quantified by OD_590_. Significance was calculated using one-way ANOVA with Tukey's (A) or Dunnett's post hoc test comparison to NI (B and D) or NT (C). **p < 0.01, ***p < 0.001, and ****p < 0.0001.

To determine the effect of LF82 infection on cell viability, T84 cells incubated with bacteria or non-treated controls were stained with Trypan blue, which penetrates dead but not live cells and is an established method to determine cell death^32^. Incubation with LF82 but not MG1655 resulted in significant cell death in confluent Caco-2 cells as well as in confluent and polarized T84 cells (Figure 5A). LF82-mediated cytotoxicity was more pronounced in confluent T84 versus Caco-2 cells and occurred as early as 1 h after gentamicin treatment, while Caco-2 cell death only became significant after 21 h post-gentamicin incubation. Interestingly, T84 cell cytotoxicity was not observed when LF82 adhesion was inhibited with mannose or when the cells were incubated with conditioned medium from LF82-infected cells (Figure 5B). In addition, the inhibition of LF82 invasion by cytochalasin D or colchicine did not significantly reduce cell death (Figure 5C). As AIEC LF82 encodes two type 6 secretion systems (T6SSs)^33^​​​​​ that might mediate the injection of toxic effector proteins into host cells, infections with LF82 mutants harboring partial deletions of the T6SS E structural units were performed. As shown in Figure 5D, the deletion of either *tssE1*, *tssE3* or both did not diminish LF82 cytotoxicity.

**Figure 6.**
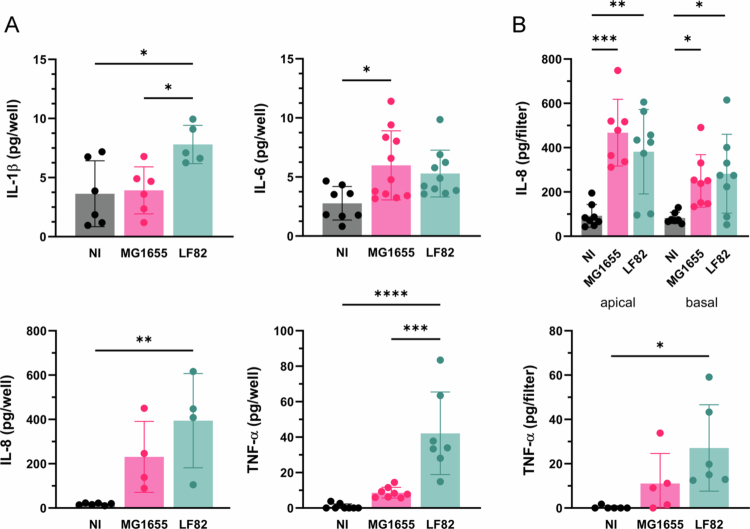
AIEC LF82 infection induces proinflammatory cytokine release. Confluent (A) and polarized T84 cells (B) were incubated with LF82, MG1655 or left noninfected (NI) for 3 h, followed by 21 h of gentamicin treatment. (A) IL-1β, IL-6, IL-8, and TNF-α levels in supernatants were quantified by ELISA. (B) IL-8 levels in apical and basal supernatants and apical TNF-α levels were quantified by ELISA. Significance was calculated using one-way ANOVA with Tukey's post hoc test comparison (*p < 0.05, **p < 0.01, ***p < 0.001, and ****p < 0.0001).

As inflammation is a predominant feature in CD, we also assessed proinflammatory cytokine production during incubation with *E. coli* MG1655 and AIEC LF82. As shown in Figure 6A, LF82 infection of confluent T84 cells significantly increased the release of IL-1β, IL-8, and TNF-α. In contrast, IL-6 expression was increased in cells incubated with both *E. coli* strains but only reached significance for MG1655. In polarized T84 cells, both LF82 and MG1655 significantly induced IL-8 secretion into the apical and basal compartments (Figure 6B). TNF-α release was only detected in apical supernatants and was significantly elevated by LF82 infection (Figure 6B). In contrast, signals for IL-1β and IL-6 remained below the detection level for all samples.

### AIEC LF82 biofilm formation and cytotoxicity are not shared by other AIEC isolates

**Figure 7.**
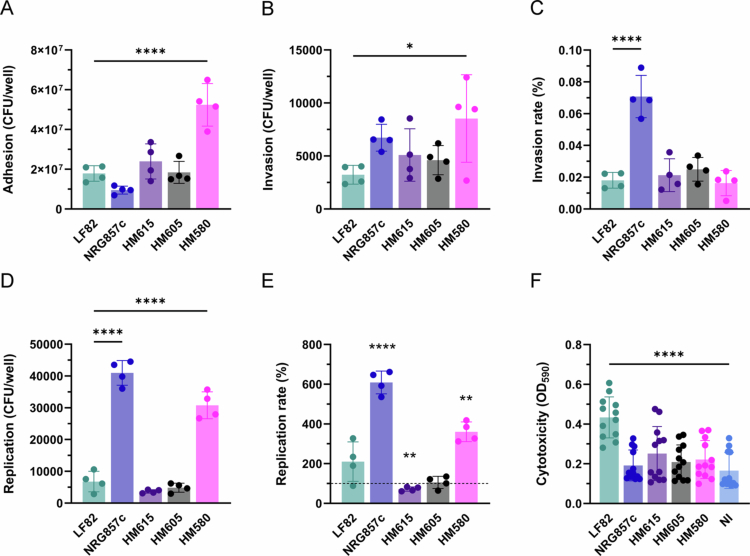
Adherence, invasion, intracellular replication and cytotoxicity of AIEC strains from CD. Confluent T84 cell monolayers were incubated with AIEC strains LF82, NRG857c, HM615, HM605, HM580, or left noninfected (NI) for 3 h, and the number of adherent bacteria was determined by CFUs (A). Invasion (B) and intracellular replication (D) were evaluated after gentamicin treatment for 1 and 21 h, respectively. The results were normalized to an inoculum of 10^7^ bacteria. Invasion (C) and replication rates (E) were calculated relative to the total number of adherent and invaded bacteria, respectively. Cytotoxicity was quantified by Trypan blue staining at OD_590_ (F). Significance was calculated using one-way ANOVA with Dunnett's post hoc test comparison to LF82 (A–E) or NI (F) (*p < 0.05 and ****p < 0.0001).

**Figure 8.**
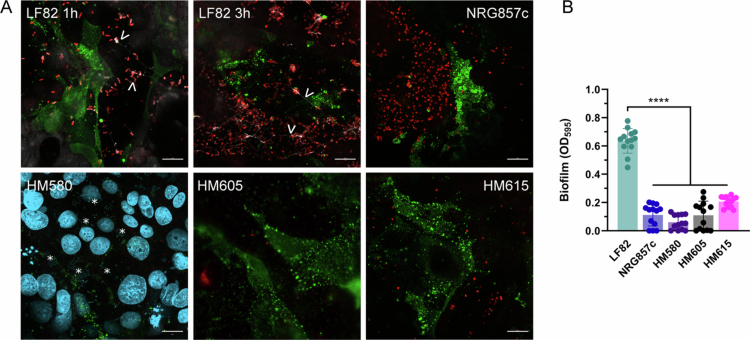
CEACAM6 binding and biofilm formation by ileal and colonic AIEC isolates. (A) Confluent T84 cells were infected with AIEC strains LF82, NRG857c, HM580, HM605, or HM615 for 1 or 3 h. Samples were stained with anti-*E. coli* (red), anti-CEACAM6 (green), calcofluor (white), and DAPI (blue, HM580 only). The cellulose fibers and DAPI-stained bacteria are labelled with arrowheads and asterisks, respectively. Representative images of three independent experiments are shown. Scale bar = 20 µm. (B) Biofilm formation on 96-well plates was quantified after 24 h by crystal violet staining. Significance was calculated using one-way ANOVA with Dunnett's post hoc test comparison to LF82 (****p < 0.0001).

We next determined whether the infection phenotype of the ileal AIEC prototype strain LF82 was exhibited by other AIEC isolates from CD patients. To this aim, we employed the ileal isolate NRG857c^34^​​​​​ and the colonic strains HM580, HM605, and HM615^21^. As shown in Figure 7A, the adhesion of strain HM580 was significantly higher compared with LF82, HM605, and HM615. In contrast, the binding of AIEC NRG857c was reduced, although this did not reach significance. Interestingly, FimH expression differed between strains with LF82 showing the highest levels and isolates HM580 and HM605 lacking type I fimbriae when incubated in cell culture medium for 3 h (Figure S2). Although the total number of invasive bacteria was significantly elevated for HM580 versus LF82 (Figure 7B), similar invasion rates were determined after normalization (Figure 7C). Notably, strain NRG857c was significantly more invasive than all other strains. Similarly, AIEC NRG857c exhibited the highest intracellular replication of all isolates, followed by HM580 (Figure 7D and E). In contrast, strains HM605 and HM615 showed no intracellular multiplication. Notably, cytotoxicity was only observed in T84 cells incubated with strain LF82 (Figure 7F). We next assessed bacterial binding to CEACAM6 and biofilm formation by immunofluorescence staining. As the *E. coli* antibody employed in this study did not recognize HM580, adherent bacteria were visualized by DNA staining with DAPI. Similar to LF82, AIEC strains NRG857c, HM580, HM605, and HM615 did not bind preferentially to CEACAM6-expressing cells (Figure 8A). While cellulose fibers were detected between adherent AIEC LF82 as early as 1 h after infection, none of the other strains showed biofilm formation. This agreed with the lack of abiotic biofilm formation in 96-well plates (Figure 8B).

### AIEC LF82 adheres to and penetrates the mucus layer of human colonoids

**Figure 9.**
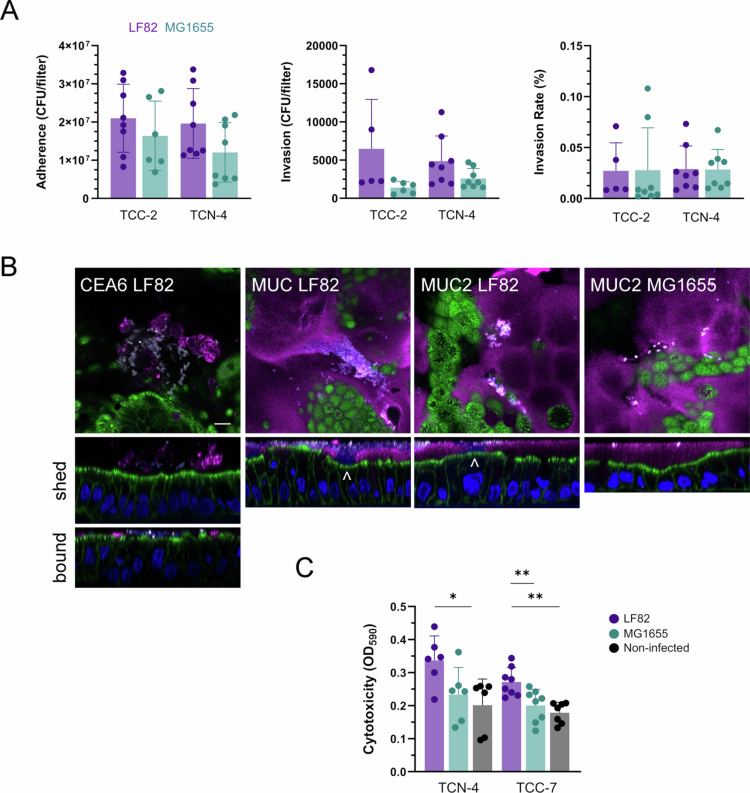
Interactions of AIEC LF82 with differentiated human colonoids. (A) Colonoid monolayers from CD (TCC-2) and non-IBD controls (TCN-4) were incubated with LF82 or MG1655 for 3 h (adhesion) followed by 1 h of gentamicin treatment (invasion), and CFUs were determined. The invasion rate was calculated as the percentage of the number of intracellular bacteria relative to adherent bacteria. Significance was calculated using student's unpaired *t*-test. (B) Colonoids from CD (TCC-2) and non-IBD controls (TCN-2 and TCN-5) cultured with LF82 or MG1655 for 3 h were stained for *E. coli* (white), CEACAM6 or MUC2 (magenta), actin (green), and DNA (DAPI, blue). Shown are merged XY (upper row) and selected XZ scans (lower rows). For CEACAM6, line scans of shed and membrane-bound receptors are shown. Arrowheads indicate penetration of DAPI-stained LF82 bacteria across the mucus layer and contact with the epithelium. Representative images of two independent experiments are shown. Scale bar = 10 µm. (C) Cytotoxicity was assessed in colonoids from CD (TCC-7) and non-IBD control (TCN-4) incubated with LF82, MG1655 or left uninfected for 3 h followed by 21 h gentamicin treatment. Trypan blue staining was quantified at OD_590_. Significance was calculated using one-way ANOVA with Tukey's post hoc test comparison (*p < 0.05 and **p < 0.01).

We next determined whether the characteristic virulence phenotype of LF82 identified in human colon carcinoma cells was reproduced in human colonoids. Similar to the results in T84 cells, the number of adherent and intracellular bacteria was higher for LF82 than MG1655, although this did not reach significance (Figure 9A). The normalized invasion rates were negligible for both strains (~0.025%) and did not differ in colonoids from CD and non-IBD controls (Figure 9A). Immunostaining of colonoids for CEACAM-6 showed lower expression than in T84 or Caco-2 cells, with most CEACAM-6 detected in extracellular vesicles (Figure 9B). Few colonocytes exhibited apical CEACAM-6 membrane staining, and there was no colocalization with adherent LF82 (Figure 9B). Interestingly, MUC2 staining indicated LF82 binding and biofilm formation in the mucus layer and penetration of bacteria to the epithelial surface (Figure 9B). In contrast, *E. coli* MG1655 remained confined to the mucus layer (Figure 9B). The levels of secreted MUC2 differed between colonoid lines and were not related to CD status (Fig. S3). Similar to results in cell lines, infection with LF82 resulted in significant cytotoxicity (Figure 9C).

## Discussion

In this study, we investigated the interaction of AIEC prototype strain LF82 with mature human colonic epithelia by using models of confluent and polarized colon carcinoma cell lines and differentiated colonoid monolayers. In contrast to early studies in subconfluent Caco-2 cells, which reported invasion rates of 4.8% relative to the initial inoculum,^5^ LF82 invasion in our colonocyte models was considerably lower (below 0.1%) and did not differ significantly from that of noninvasive *E. coli* MG1655. This discrepancy is likely due to established cell polarity and the formation of tight junctions, which segregate apically and basolaterally expressed cell surface proteins and thereby restrict bacterial binding receptors accessible via the apical side.^33^ This is in line with the lower invasion of AIEC strain NRG857c in polarized versus non-polarized Caco−2 cells.^34^ Notably, an invasion rate of 0.013% in polarized Caco-2 cells was comparable to the results of our study. Several intestinal pathogens, including *Listeria monocytogenes* and *Shigella flexneri* bind to basolateral membrane receptors after penetrating the epithelium via M cells or disrupted tight junctions.^35^^,^^36^ However, AIEC LF82 does not show preferential adhesion to the basolateral cell surface. In addition to the lack of invasion, LF82 biofilm formation is a distinctive feature on human colonic epithelia. This finding agrees with previous studies demonstrating higher biofilm formation by AIEC than other mucosa-associated *E. coli* strains^10​​​​^ and cellulose production in AIEC biofilms.^37^ Interestingly, the mucosal biofilm formation and colonization of mice by AIEC NRG857c has been attributed to a type IV secretion system. However, the corresponding *tra* locus is absent in strain LF82.^38^ In contrast, the ileal AIEC strain NRG857c and colonic CD isolates HM580, 605, and 615 exhibited no or lower biofilm formation on human colonic epithelia, which correlates with previous studies on abiotic surfaces.^39^ Given the high sequence similarity between strains LF82 and NRG857c^40^, their difference in biofilm production is surprising. However, recent studies identified a rare single nucleotide polymorphism in the LF82 RNA polymerase sigma factor σ70, which results in transcriptomic and phenotypic changes, including increased biofilm formation.^41^ As this variant was not found in other AIEC strains, this might explain the exceptional biofilm production by AIEC LF82.

Another unexpected finding of this study was the independence of AIEC epithelial binding of CEACAM6. While we confirmed that LF82 expressed type I fimbriae and adhesion was inhibited by mannose, as shown previously,^7^ no preferential bacterial binding was observed to CEACAM6-rich cells, and CEACAM6-specific antibodies did not reduce adherence. This finding contrasts with previous work that demonstrated CEACAM6-specific binding of AIEC LF82 to ileal enterocytes from CD patients.^7^ In addition, AIEC LF82 induced colitis in CEABAC10 transgenic mice harboring human CEACAM3, CEACAM5, CEACAM6, and CEACAM7 genes, and intraperitoneal injection of anti-CEACAM6 significantly decreased LF82 colonization.^18^ As the same antibody (mouse monoclonal 9A6) was used for CEACAM6 blocking in all studies, LF82 might engage with different host receptors in human colonic epithelial cells. CEACAM6 is an attractive AIEC receptor candidate in the small intestine as its expression is induced by inflammation. Interestingly, higher CEACAM6 levels were detected in ileal but not colonic tissue from CD versus non-IBD control tissues, thereby indicating higher susceptibility of ileal CD mucosa to AIEC binding.^7^^,^^15^ In addition, AIEC translocation across the follicle-associated epithelium in ileal Peyer's patches has been attributed to binding of long polar fimbriae to CEACAM6.^12^^,^^42^ Nevertheless, adherence of LF82 to ileal and colonic biopsy samples from CD patients and healthy controls did not differ significantly.^43^

Another reason for the lack of colonic CEACAM6 binding by LF82 could be the strain's adaptation to its original colonization site (i.e. the ileum). Therefore, colonic AIEC isolates (HM580, HM605, and HM615) were examined, but there was no preferential binding to CEACAM6-rich cells. Interestingly, FimH expression in the ileal and colonic AIEC isolates did not correlate with adhesion. Despite lacking type I fimbriae, strain HM580 demonstrated higher binding than all other isolates suggesting the involvement of other adhesins, including ChiA and OmpA.^8^^,^^9^ In addition to the lack of biofilm formation discussed above, isolates HM580, HM605, HM615, and NRG857c differed from LF82 in that they did not cause cell death. Notably, these two features could be related, as biofilms enable close proximity of high bacterial numbers to the cell surface and thereby promote efficient toxin delivery.^44^ Interestingly, previous work has demonstrated that fragmentation of the mitochondrial network in LF82-infected T84 cells leads to increased epithelial permeability and DNA breakage, which are indicative of cell death.^45^ Similar to our findings, this effect was not mediated by spent culture medium, thereby ruling out secreted virulence factors unless very unstable. Notably, genome sequencing of LF82 has identified two T6SSs (T6SS-1 and T6SS-3) located on pathogenicity islands PAI-1 and PAI-3, respectively.^46^ Although no effector proteins and conclusive functions have been assigned to LF82 T6SS-1 and T6SS-3 so far, T6SSs of Gram-negative bacteria have been shown to deliver toxins into other bacteria and also eukaryotic host cells.^47^ In particular, the antimicrobial T6SS effector VgrG4 from *Klebsiella pneumoniae* triggered mitochondrial network fragmentation in lung epithelial cells; therefore, arole of the LF82 T6SS in colonic epithelial cytotoxicity would be plausible.^48^ However, no reduced cytotoxicity was detected in infections with T6SS-deficient mutants in our study, indicating other mechanisms of contact-dependent cytotoxicity. In addition to cell death, LF82 infection resulted in secretion of proinflammatory cytokines IL-1β, IL-6, IL-8, and TNF-α*,* indicating a causative or at least exacerbating role of AIEC in CD. This finding is in accordance with previous work demonstrating pro-inflammatory cytokine secretion in AIEC-infected mice and human colonic cell lines.^16–18^

While most characteristics of LF82 infection in T84 cells were mirrored in human colonoids, including biofilm formation, lack of invasion and cytotoxicity, bacteria adhered predominantly to the colonoid mucus layer rather than any exposed epithelium. As shown previously, CEACAM6 expression in human colonic epithelium was lower than that in the Caco-2 and T84 cell lines.^7^ Although there was colocalization of LF82 with CEACAM6, this was restricted to CEACAM6 released from the cell membrane. Interestingly, a recent study demonstrated that CEACAM6-laden extracellular vesicles from enterocytes acted as a decoy for ETEC and its heat-labile toxin and enhanced bacterial clearance from the gut.^49^ While we did not observe binding of LF82 to CEACAM6 at the apical cell membrane, bacteria were predominantly associated with the overlying mucus layer. Notably, early in situ analyses of mucosa-associated *E. coli* in the ileal tissue of CD patients showed prevalent bacterial localization in the superficial mucus.^50^ AIEC mucus binding might be mediated by flagella or fimbriae, as shown for attaching-effacing *E. coli* ^51^ and ETEC,^52^ respectively. In addition to binding, penetration of the mucus layer was evident for AIEC LF82 but not *E. coli* MG1655. This could be mediated by the serine protease autotransporter Vat, which has been shown to degrade intestinal mucus and thereby promote AIEC mucosal colonization in mice.^53^ Furthermore, a recent study has implicated the AIEC chitin-binding protein ChiA in intestinal mucus binding and degradation.^54^ Preferential AIEC LF82 mucus binding in colonoids also agrees with higher adhesion to mucus-producing LS174T cells versus mucus-deficient Caco-2 and T84 cells. However, this might also be due to the lack of LS174T cell polarity and the availability of additional binding receptors.

Interestingly, previous research on the interaction of AIEC with human sigmoid colonoids reported higher numbers of intracellular bacteria for LF82 compared with *E. coli* K-12 relative to the initial inoculum.^55^ This is consistent with our results on invasion without accounting for differences in adhesion. However, LF82 invasion in the earlier study was considerably higher with 1.14% of the inoculum surviving gentamicin treatment compared with 0.06% in our work. This can likely be attributed to a higher multiplicity of infection (20 bacteria/cell) and longer incubation period before gentamicin treatment (6 h). In addition, colonoid monolayers might be less polarized due to culture on nonpermeable surfaces and only 2 d of differentiation.^55^

Notably, LF82 infection resulted in a similar phenotype in colonoids derived from CD patients and non-IBD controls. This is unexpected, as reduced goblet cell numbers and MUC2 secretion have been reported in CD tissue,^56^^,^^57^ which would likely promote AIEC LF82 adhesion to the epithelium and associated cell damage. However, our results show that MUC2 production in colonoids is independent of CD status, although this is limited to only four colonoid lines investigated. While intestinal organoids derived from adult stem cells are genetically and epigenetically stable^58^^,^^59^, a loss of inflammatory signature has been observed in colonoids from patients with ulcerative colitis.^60^ It remains to be investigated whether colonoids maintain mucus secretion comparable to that of original tissue samples.

In summary, we have demonstrated that infection of differentiated human colonic epithelial cells by AIEC LF82 is characterized by biofilm formation, mucus penetration, and contact-dependent cytotoxicity. Therefore, future research should focus on strategies aimed at biofilm disruption and mucinase inactivation and identify LF82 effectors that mediate cell death. In addition, the virulence mechanisms of other AIEC isolates need to be elucidated in differentiated intestinal cell models.

## Supplementary Material

Supplementary material Figure S1: CEACAM6-specific antibodies reduce the binding of ETEC but not AIEC LF82 to T84 cells. Cells preincubated with mouse or rabbit antibodies against CEACAM6 (mCEA6, rbCEA6) or left untreated (NT) were infected with LF82 or ETEC strain H10407 for 3 h, and adhesion was quantified by CFUs. Mannose (man) was included to block fimbrial binding. Significance was calculated using one-way ANOVA with Dunnett's posttest comparison with the NT (*p < 0.05, **p < 0.01, ***p < 0.001, ****p < 0.0001).

Supplementary material Figure S2: AIEC isolates differ in FimH expression. Bacteria cultured in DMEM/F-12 medium for 3 h were serially diluted at ratios of 1:2–1:32. The production of type I fimbriae was determined by yeast agglutination. The red circles indicate the highest bacterial dilution resulting in yeast agglutination. A representative image of *n* = 3 samples is shown.

Supplementary material Figure S3: MUC2 secretion by colonoids is donor-specific. Colonoid monolayers from CD (TCC-6 and TCC-7) and non-IBD controls (TCN-1 and TCN-2) were stained for MUC2 and cell nuclei (DAPI). Scale bar = 50 µm (A). MUC2 levels were quantified by integrated density (B).

## Data Availability

The authors confirm that the data supporting the findings of this study are available within the article and its supplementary materials.
