# Supplementary figures and images for: Pathogenesis of adherent-invasive Escherichia coli LF82 in human colonic epithelium is characterized by adhesive biofilms, mucus penetration, and contact-dependent cytotoxicity
Source: Gut Microbes. 2025 Oct 29;17(1):2573046. doi: 10.1080/19490976.2025.2573046 (PMC12578305; doi:10.1080/19490976.2025.2573046)

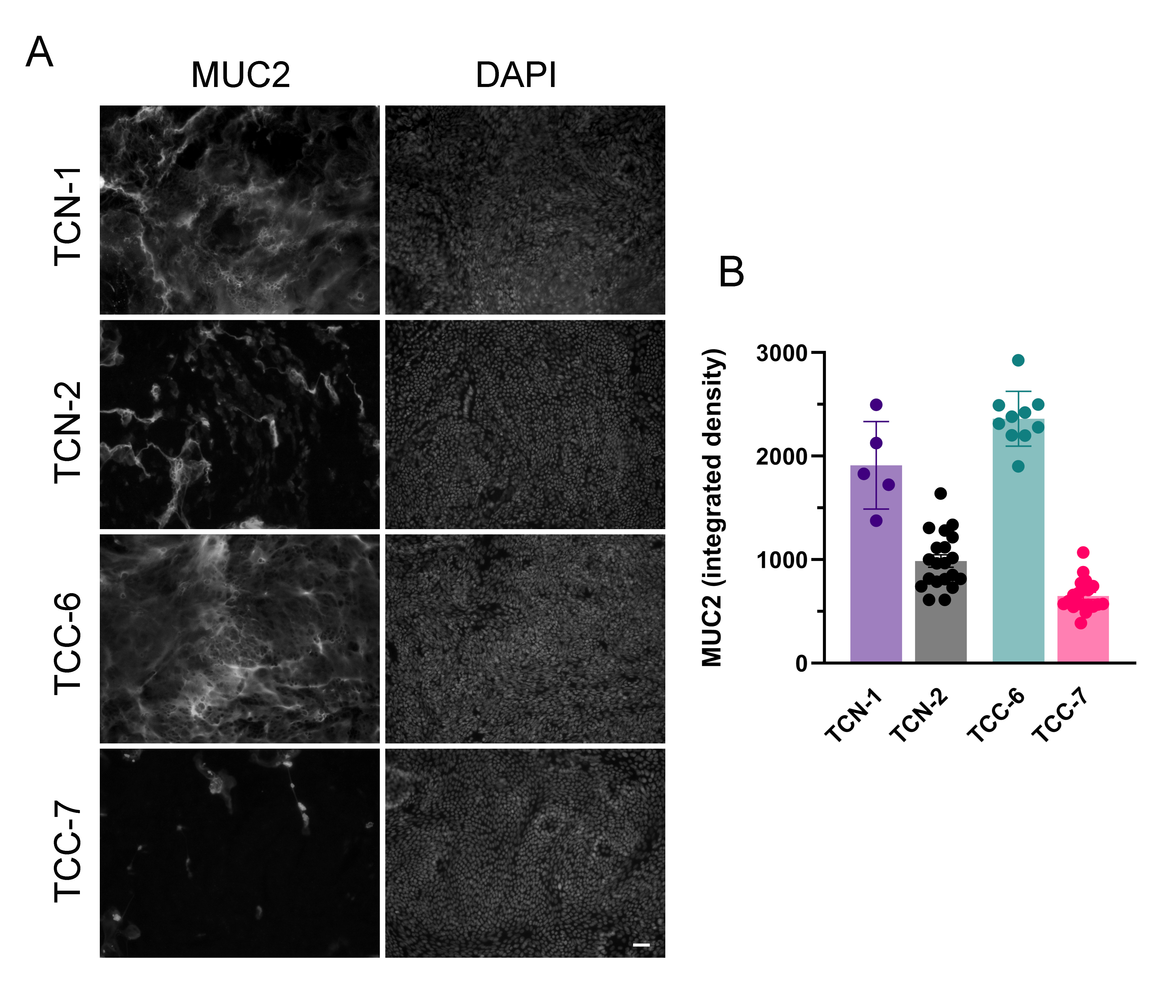

Supplement: Supplementary material — Figure S1: CEACAM6-specific antibodies reduce the binding of ETEC but not AIEC LF82 to T84 cells. Cells preincubated with mouse or rabbit antibodies against CEACAM6 (mCEA6, rbCEA6) or left untreated (NT) were infected with LF82 or ETEC strain H10407 for 3 h, and adhesion was quantified by CFUs. Mannose (man) was included to block fimbrial binding. Significance was calculated using one-way ANOVA with Dunnett's posttest comparison with the NT (*p < 0.05, **p < 0.01, ***p < 0.001, ****p < 0.0001). [file KGMI_A_2573046_SM0500.tif]

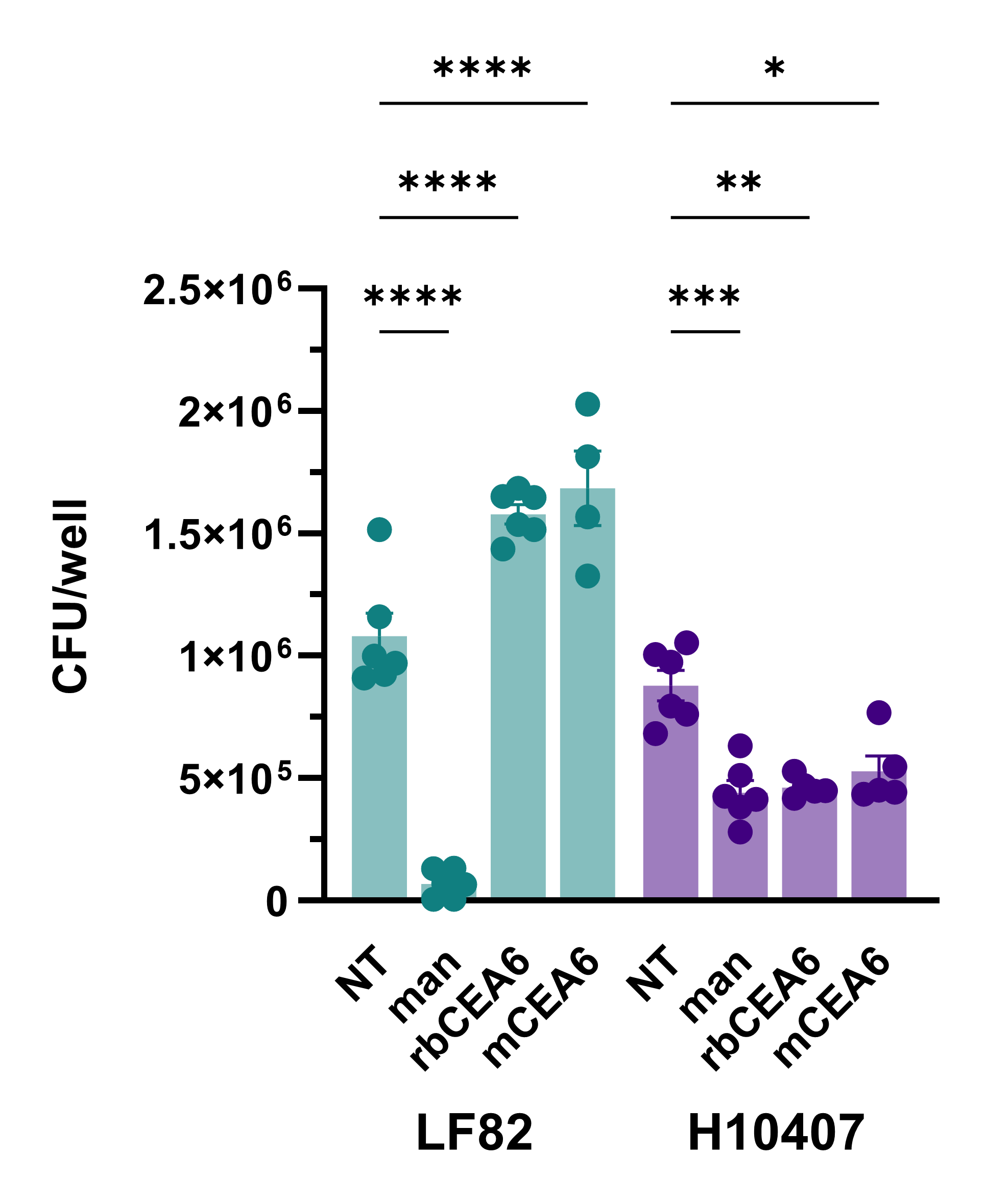

Supplement: Supplementary material — Figure S2: AIEC isolates differ in FimH expression. Bacteria cultured in DMEM/F-12 medium for 3 h were serially diluted at ratios of 1:2–1:32. The production of type I fimbriae was determined by yeast agglutination. The red circles indicate the highest bacterial dilution resulting in yeast agglutination. A representative image of n = 3 samples is shown. [file KGMI_A_2573046_SM0499.tif]

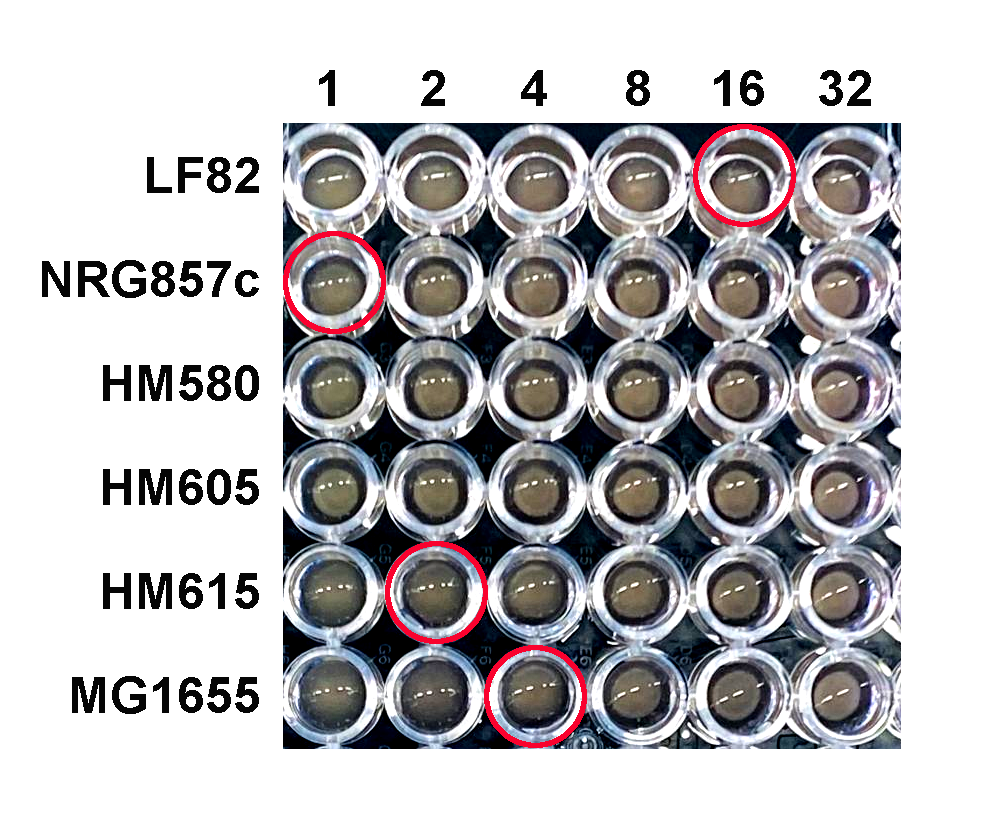

Supplement: Supplementary material — Figure S3: MUC2 secretion by colonoids is donor-specific. Colonoid monolayers from CD (TCC-6 and TCC-7) and non-IBD controls (TCN-1 and TCN-2) were stained for MUC2 and cell nuclei (DAPI). Scale bar = 50 µm (A). MUC2 levels were quantified by integrated density (B). [file KGMI_A_2573046_SM0498.tif]
